# HPV genotypes in invasive cervical cancer: prevalence, risk attribution, and optimized vaccine strategies in western China

**DOI:** 10.3389/fpubh.2024.1455931

**Published:** 2024-12-18

**Authors:** Yuling Kou, Xiao Tang, Dongni Liang, Chuan Xie, Jing Zeng, Meng Chen, Wenjing Fu, Zhonghua Li, Qingfeng He, Tianming Liu, Mei Wang, Wei Wang, Cheng Wang

**Affiliations:** ^1^Department of Pathology, West China Second University Hospital, Sichuan University, Chengdu, China; ^2^Key Laboratory of Birth Defects and Related Diseases of Women and Children, Ministry of Education, Chengdu, China; ^3^Department of Gynecology and Obstetrics, West China Second University Hospital, Sichuan University, Chengdu, China

**Keywords:** invasive cervical cancer, genotypes, human papillomavirus, infection, vaccine

## Abstract

**Background:**

Understanding the HPV genotype distribution in invasive cervical cancer (ICC) is essential for vaccine optimization. This study presents a comprehensive analysis of HPV genotypes in ICC tissues from patients in western China, with the aim of informing regional vaccine policy and prevention strategies.

**Methods:**

DNA was extracted from 1,908 paraffin-embedded ICC samples, and 23 HPV genotypes were detected via PCR and reverse dot hybridization gene chip assays. The genotypic distribution of HPV infections was analyzed, the attribution of each HPV genotype found in multiple infection cases was calculated using the fractional contribution approximation. Furthermore, the cumulative attribution rates of HPV genotypes included in each vaccine combination were totaled to estimate the potential vaccination coverage of ICC across various histologic types and age groups.

**Results:**

The overall prevalence of HPV infection was 94.9% (95% CI 93.8–95.8) among 1,908 women with ICC. HPV genotypes 16 and 18 were detected in 1645 of 1810 HPV-positive patients (90.9, 95% CI 89.5–92.1) of ICC. HPV16, 18, 33, 52, and 58 were detected in 1,749 patients (96.6, 95% CI 95.7–97.4), the five most common genotypes in different age groups. HPV genotypes contained in the 9-valent vaccine were detected in 1776 patients (98.1, 95% CI 97.4–98.7). By weighted imputation analysis, the cumulative attribution rates of the bivalent vaccine was 83.4%, and that of the nine-valent vaccine was 89.8%. Optimization group A included the five genotypes with the highest prevalence, HPV16, 18, 33, 52, and 58, with a cumulative attribution rates of 88.5%, and optimization group B included the nine most common HPV genotypes, HPV16, 18, 31, 33, 35, 45, 52, 58, and 59, with a cumulative attribution rates of 90.5%.

**Conclusion:**

Our comprehensive postsurgical analysis of HPV in ICC patients in western China revealed that the incorporation of the bivalent vaccine into the national program is cost-effective, with group A optimization closely matching the vaccination coverage of the 9-valent vaccine, which can be used to guide future prevention strategies.

## 1 Introduction

Invasive cervical cancer (ICC) is the fourth most common cancer in adult females globally and the second most common cancer in females between the ages of 15 and 44 (1). It is estimated that more than 58% of global ICC incidence and deaths occur in Asia, with 18% of ICC incidence and 17% of deaths occurring in China (2). Persistent infection with human papillomavirus (HPV) is a major causative factor of cervical cancer development; it is associated with more than 90% of cervical tumors (3). However, the vast majority of asymptomatic HPV infections can be cleared briefly by natural defenses (4). HPV16 and HPV18 are the most notable and prevalent carcinogenic genotypes, accounting for more than 70% of carcinomas of the uterine cervix worldwide (5).

At present, more than 200 HPV genotypes have been identified, and according to their capacity for viral malignant transformation, they have been classified as high-risk HPV (HR-HPV) or low-risk HPV (LR-HPV) (6, 7). There is regional or ethnic diversity in the distribution of HPV prevalence and genotypes, which may affect the effectiveness prevention strategies, creating challenges for HPV-related disease management. The ultimate goal of HPV vaccination is to prevent ICC by preventing infection with significant cancer-causing HPV genotypes (8). Bivalent, quadrivalent, and nine-valent vaccines are the most widely used vaccines worldwide. Moreover, no vaccine can prevent all genotypes of HPV infections. Therefore, combining the distribution of HPV genotypes and formulating an HPV vaccine that best matches the geographic distribution can maximize cervical cancer prevention while maximizing cost-effectiveness and affordability.

Population-based HPV genotyping data of ICC patients will establish a baseline of the HPV genotype-specific disease burden as a basis for optimizing ICC prevention strategies. Systematic evaluation of the age distribution of HPV genotypes, histological types distribution, and attribution rate in paraffin-embedded tissues from large samples of patients with ICC in western China is lacking. In the present study, we investigated a large population of ICC patients in western China. We obtained comprehensive information about the distribution of HPV genotypes and evaluated the associations between tumor HPV status, tumor histological types, and age. Such knowledge is essential for evaluating the potential impact, cost-effectiveness, and strategic deployment of HPV vaccines. These are crucial tools in ICC prevention programs, offering insights to guide vaccine policy and prevention efforts in this understudied region.

## 2 Materials and methods

### 2.1 Participant enrollment and data collection

In this retrospective study, we estimated the prevalence of HPV genotypes in formalin-fixed paraffin-embedded (FFPE) samples from women who received primary radical treatment and were diagnosed with ICC between January 2018 and December 2023 at the Department of Pathology, West China Second University Hospital, Sichuan University (WCSUH), which is the largest medical center serving a large number of patients in western China. We reviewed the clinical records associated with all samples with information about the original histological diagnosis and other essential information. The inclusion criterion was patients in 12 provinces in western China, including Sichuan, Tibet, Yunnan, Guizhou, Xinjiang, Gansu, Shanxi, Guangxi, Chongqing, Qinghai, Inner Mongolia, and Ningxia, who underwent radical hysterectomy, and FFPE tumor samples from patients who were diagnosed with primary ICC were selected by experienced pathologists for HPV DNA testing. We excluded FFPE samples from other hospitals that were submitted for testing and those from patients with metastatic cervical cancer from this study. FFPE samples were obtained from pathology archives for HPV genotyping after the study protocol was approved by the Ethics Committee of WCSUH (Approval No.2024 (119)). Patient data and samples were completely anonymized.

### 2.2 HPV DNA extraction and HPV genotype testing

All samples were sectioned at the Molecular Pathology Laboratory at WCSUH. Paraffin blocks were selected from identified tumor samples for hematoxylin and eosin staining of the first and fourth 4-μm-thick tissue sections for histopathological diagnosis; the second and third sections were used for HPV DNA genotyping via the sandwich method. Blank paraffin sections were cut to prevent contamination between tumor specimens. Samples greater than 2 cm^2^ and containing a minimum of 70% tumor cells were chosen to ensure the accuracy of the data for each block. Curling sections were deparaffinized with xylene in 1.5-mL Eppendorf tubes, and genomic DNA was isolated using a QIAamp^®^ DNA FFPE Tissue Kit (Qiagen. Hilden, Germany) according to the manufacturer’s instructions. DNA extraction was performed using a PCR-RDB HPV genotyping kit (Yaneng^®^ Limited Corporation, Shenzhen, China) (9). This method can be used to test for 17 high-risk HPV genotypes (16, 18, 31, 33, 35, 39, 45, 51, 52, 53, 56, 58, 59, 66, 68, 73, and 82) and six low-risk HPV genotypes (6, 11, 42, 43, 81, and 83). PCR amplification, hybridization, incubation, and color development were carried out according to the manufacturer’s protocols. Finally, positive results appeared as a blue dot observable by the naked eye on the strip, and the amplification of the human *β*-globin gene was used as an internal control for each sample.

### 2.3 Statistical analysis

Descriptive statistical analyses were also conducted. The overall prevalence rate was defined as the total number of positive samples divided by the total number of samples tested in that category. For the relative distribution, only HPV-positive samples were used as the denominator. The variables analyzed included age at diagnosis, histopathological diagnosis, and HPV genotype. The fractional contribution approximation/weighted imputation analysis method, as previously described in the literature (10), posits that each HPV genotype within a coinfection exerts a distinct degree of influence on the development of cervical cancer. Consequently, when more than one HPV-genotype is detected in a sample, multiple infections are weighted toward a single type. Calculated as follows: prevalence of single-type infections + (prevalence of multiple-type infections × attribution factor). The attribution factor is calculated as the number of samples with single-type infection by the relevant HPV genotype divided by the number of samples with any HPV genotype single-type infection within the same disease category (11). The cumulative attribution rate is the sum of the individual attribution rates for each HPV genotype in the vaccine combination, which we use to indicate potential vaccination coverage. All the statistical analyses were performed using R software (version 4.2.1). Categorical variables were compared using Pearson chi-square tests to evaluate the significance of the comparisons. The 95% confidence interval (CI) for proportions was calculated using Wilson’s criteria. The collected data were processed and visualized using GraphPad Prism 8 (GraphPad Software, San Diego, United States).

## 3 Results

### 3.1 Study population

**Figure 1:**
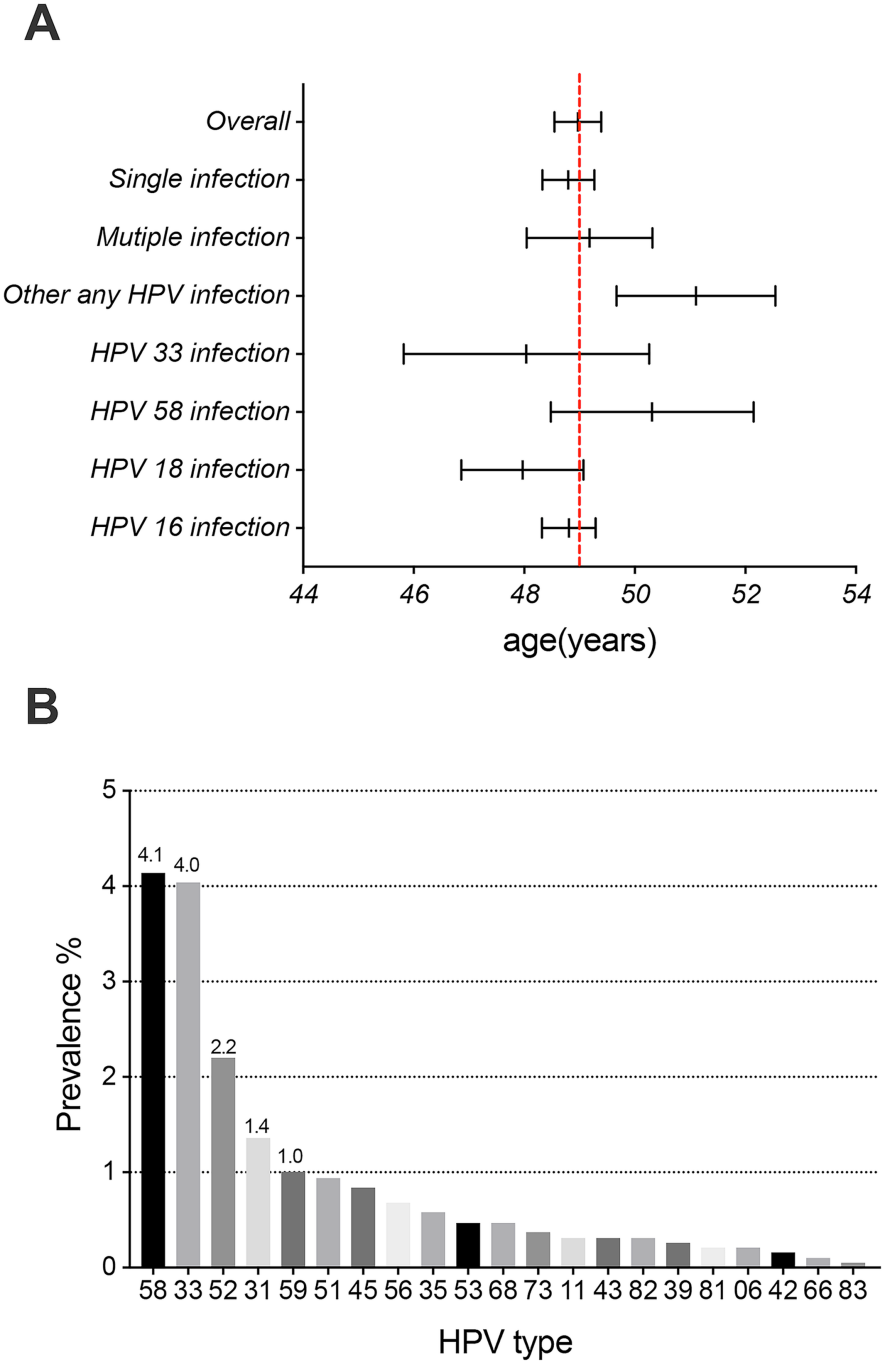
Prevalence and mean age (95% CI) at diagnosis of invasive cervical cancer. **(A)** Overall: 48.97 years (95% CI, 48.55–49.40), HPV16 infection: 48.81 years (95% CI, 48.32–49.29), HPV 18 infection: 47.97 years (95% CI, 46.86–49.07), HPV58 infection: 50.32 years (95% CI, 48.49–52.15), HPV33 infection: 48.04 years (95% CI, 45.82–50.26), other HPV infection: 51.11 years (95% CI, 49.67–52.55), single HPV infection: 48.80 years (95% CI, 48.33–49.27), and multiple HPV infection: 49.18 years (95% CI, 48.04–50.32). **(B)** The prevalence of 21 HPV genotypes (excluding HPV genotypes 16 and 18) in invasive cervical cancer patients.

After histological evaluation of FFPE tissues archived between 2018 and 2023, HPV testing results of tumor tissues from 1,908 female patients with ICC from 12 provinces in western China (with the greatest number from Sichuan Province, Supplementary Figure S1) were obtained in this study, and the samples included those diagnosed with squamous cell carcinoma (SCC, *n* = 1,436), adenocarcinoma (ADC, *n* = 308), adenosquamous cell carcinoma (ADSQ, *n* = 129) and other rare carcinomas (*n* = 35, including neuroendocrine carcinoma, carcinosarcoma, and adenoid basal carcinoma). The overall prevalence of HPV infection in the 1908 patients with ICC was 94.9% (1,810/1,908), the single infection rate was 80.2%, and the multiple infection rate was 14.7%. There were 1,270 patients with HPV-positive ICC in the 40–59 age group, accounting for 70.2% of the overall positive patients (Table 1). The mean age of HPV-positive and negative patients was 48.9 years and 51.1 years, respectively. Additionally, the mean age of patients infected with HPV16, 18, and 33 was lower than the cohort’s average. Furthermore, the mean age for patients with single infections was lower than that for those with multiple infections (Figure 1A). Overall infection rates ≥1% for all HPV genotypes, including HPV 16, 18, 58, 33, 52, 31, and 59 (Figure 1B).

**Table 1:** Characteristics of study subjects.

	HPV negative (*N* = 98, 5.1%)	HPV positive (*N* = 1,810, 94.9%)	Overall (*N* = 1,908, 100%)	*p* value
**Age group**				0.351
20–29y	0 (0%)	31 (1.7%)	31 (1.6%)	
30–39y	13 (13.3%)	275 (15.2%)	288 (15.1%)	
40–49y	34 (34.7%)	626 (34.6%)	660 (34.6%)	
50–59y	32 (32.7%)	644 (35.6%)	676 (35.4%)	
≥60 y	19 (19.4%)	234 (12.9%)	253 (13.3%)	
**Age**				0.074
Mean (SD)	51.1 (10.3)	48.9 (9.42)	49.0 (9.48)	
Median [Min, Max]	51.0 [31.0, 86.0]	49.0 [21.0, 77.0]	49.0 [21.0, 86.0]	
**Histology**				<0.001
SCC	45 (45.9%)	1,391 (76.9%)	1,436 (75.3%)	
ADC	39 (39.8%)	269 (14.9%)	308 (16.1%)	
ADCA	7 (7.1%)	122 (6.7%)	129 (6.8%)	
Others	7 (7.1%)	28 (1.5%)	35 (1.8%)	

HPV, human papillomavirus; SCC, squamous cell carcinoma; ADC, adenocarcinoma; ADSQ, adenosquamous cell carcinoma; Others, other rare carcinomas.

### 3.2 Relative distribution of human papillomavirus genotypes in patients with different histological types of ICC

All ICC patients in our study cohort were categorized into four histological types. These patients exhibited single and multiple infections with 17 HR-HPV genotypes, whereas 6 LR-HPV genotypes were detected solely in the context of multiple infections within SCC and ADC cases (Supplementary Table S1). HPV16, 18, 33, and 58 were the most common genotypes in HPV-positive SCC and ADC samples. Moreover, HPV52, 31, and 51 in SCC and HPV 56, 35, and 45 in ADC were also common. HPV16 and 18 were the most common genotypes in HPV-positive ADSQ and other rare carcinomas; HPV59, 45, 51, 52, and 58 in ADSQ and HPV33, 52, 58, and 59 in other histologic types were also common (Table 2).

**Table 2:** Relative distribution of the seven most common HPV genotypes observed in different histologic types of HPV-positive invasive cervical cancer.

	SCC (*n* = 1,391)			ADC (*n* = 269)			ADSQ (*n* = 122)			Others (*n* = 28)		
	Single # (%)	≥2 types # (%)	Total # (%)	Single # (%)	≥2 types # (%)	Total # (%)	Single # (%)	≥2 types # (%)	Total # (%)	Single # (%)	≥2 types # (%)	Total # (%)
HPV16	1,008 (72.5)	185 (13.3)	1,193 (85.8)	148 (55.0)	36 (13.4)	184 (68.4)	54 (44.3)	25 (20.5)	79 (64.8)	6 (21.4)	7 (25.0)	13 (46.4)
HPV18	41 (2.9)	70 (5.0)	111 (8.0)	72 (26.8)	36 (13.4)	108 (40.1)	35 (28.7)	14 (11.5)	49 (40.2)	13 (46.4)	5 (17.9)	18 (64.3)
HPV33	38 (2.7)	35 (2.5)	73 (5.2)	2 (0.7)	1 (0.4)	3 (1.1)	0 (0.0)	0 (0.0)	0 (0.0)	1 (3.6)	0 (0.0)	1 (3.6)
HPV58	33 (2.4)	38 (2.7)	71 (5.1)	1 (0.4)	3 (1.1)	4 (1.5)	0 (0.0)	3 (2.5)	3 (2.5)	0 (0.0)	1 (3.6)	1 (3.6)
HPV52	18 (1.3)	18 (1.3)	36 (2.6)	1 (0.4)	1 (0.4)	2 (0.7)	0 (0.0)	3 (2.5)	3 (2.5)	1 (3.6)	0 (0.0)	1 (3.6)
HPV31	14 (1.0)	10 (0.7)	24 (1.7)	0 (0.0)	0 (0.0)	0 (0.0)	1 (0.8)	1 (0.8)	2 (1.6)	0 (0.0)	0 (0.0)	0 (0.0)
HPV51	0 (0.0)	14 (1.0)	14 (1.0)	0 (0.0)	1 (0.4)	1 (0.4)	1 (0.8)	2 (1.6)	3 (2.5)	0 (0.0)	0 (0.0)	0 (0.0)
HPV45	8 (0.6)	3 (0.2)	11 (0.8)	1 (0.4)	1 (0.4)	2 (0.7)	1 (0.8)	2 (1.6)	3 (2.5)	0 (0.0)	0 (0.0)	0 (0.0)
HPV35	5 (0.4)	4 (0.3)	9 (0.6)	0 (0.0)	2 (0.7)	2 (0.7)	0 (0.0)	0 (0.0)	0 (0.0)	0 (0.0)	0 (0.0)	0 (0.0)
HPV56	2 (0.1)	7 (0.5)	9 (0.6)	0 (0.0)	3 (1.1)	3 (1.1)	0 (0.0)	1 (0.8)	1 (0.8)	0 (0.0)	0 (0.0)	0 (0.0)
HPV59	5 (0.4)	4 (0.3)	9 (0.6)	1 (0.4)	1 (0.4)	2 (0.7)	2 (1.6)	5 (4.1)	7 (5.7)	0 (0.0)	1 (3.6)	1 (3.6)

#, number; HPV, human papillomavirus; SCC, squamous cell carcinoma; ADC, adenocarcinoma; ADSQ, adenosquamous cell carcinoma; Others, other rare carcinomas. Cases in which multiple HPV genotypes were detected were counted for each genotype, and so were counted more than once.

### 3.3 Relative distribution of HPV infection in patients with ICC in different age groups

All patients were categorized into age groups: 20–29 years, 30–39 years, 40–49 years, 50–59 years, and 60 years and older. HPV was detected in all ICC patients aged 20–29 years, among whom the youngest patient was 21 years old. Among all age groups, the incidence of HPV single infections was lowest in patients ≥60 years of age and highest in patients aged 20–29 years (Supplementary Table S2). The prevalence of HPV16 was essentially the same in each age group, whereas that of HPV18 showed a slowly decreasing trend. HPV16, 18, 33, 52, and 58 were the most common genotypes in HPV-positive patients aged 30 years and older. The HPV genotypes that followed closely in prevalence at ages 30–49 years were HPV51 and 31; at 50–59 years, they were HPV31 and 45; and at ages ≥60, they were HPV31 and 56. Moreover, the most common HPV genotypes in 20–29-year-old patients were HPV 16, 18, 35, 33, and 58 (Table 3).

**Table 3:** Relative distribution of the seven most common HPV genotypes observed in HPV-positive ICC patients in different age groups.

	20–29y (*n* = 31)			30–39y (*n* = 275)			40–49y (*n* = 626)			50–59y (*n* = 644)			≥60 y (*n* = 234)		
	Single # (%)	≥2 types # (%)	Total # (%)	Single # (%)	≥2 types # (%)	Total # (%)	Single # (%)	≥2 types # (%)	Total # (%)	Single # (%)	≥2 types # (%)	Total # (%)	Single # (%)	≥2 types # (%)	Total # (%)
HPV16	22 (71.0)	3 (9.7)	25 (80.6)	187 (68.0)	43 (15.6)	230 (83.6)	421 (67.3)	81 (12.9)	502 (80.2)	434 (67.4)	88 (13.7)	522 (81.1)	152 (65.0)	38 (16.2)	190 (81.2)
HPV18	5 (16.1)	2 (6.5)	7 (22.6)	25 (9.1)	25 (9.1)	50 (18.2)	63 (10.1)	38 (6.1)	101 (16.1)	52 (8.1)	43 (6.7)	95 (14.8)	16 (6.8)	17 (7.3)	33 (14.1)
HPV31	0 (0.0)	0 (0.0)	0 (0.0)	2 (0.7)	1 (0.4)	3 (1.1)	5 (0.8)	5 (0.8)	10 (1.6)	6 (0.9)	4 (0.6)	10 (1.6)	2 (0.9)	1 (0.4)	3 (1.3)
HPV33	0 (0.0)	1 (3.2)	1 (3.2)	6 (2.2)	10 (3.6)	16 (5.8)	16 (2.6)	9 (1.4)	25 (4.0)	12 (1.9)	12 (1.9)	24 (3.7)	7 (3.0)	4 (1.7)	11 (4.7)
HPV35	1 (3.2)	0 (0.0)	1 (3.2)	0 (0.0)	2 (0.7)	2 (0.7)	1 (0.2)	3 (0.5)	4 (0.6)	2 (0.3)	0 (0.0)	2 (0.3)	1 (0.4)	1 (0.4)	2 (0.9)
HPV52	0 (0.0)	0 (0.0)	0 (0.0)	2 (0.7)	4 (1.5)	6 (2.2)	5 (0.8)	6 (1.0)	11 (1.8)	9 (1.4)	5 (0.8)	14 (2.2)	4 (1.7)	7 (3.0)	11 (4.7)
HPV58	0 (0.0)	1 (3.2)	1 (3.2)	3 (1.1)	2 (0.7)	5 (1.8)	12 (1.9)	21 (3.4)	33 (5.3)	14 (2.2)	13 (2.0)	27 (4.2)	5 (2.1)	8 (3.4)	13 (5.6)
HPV51	0 (0.0)	0 (0.0)	0 (0.0)	0 (0.0)	5 (1.8)	5 (1.8)	0 (0.0)	8 (1.3)	8 (1.3)	1 (0.2)	3 (0.5)	4 (0.6)	0 (0.0)	1 (0.4)	1 (0.4)
HPV59	0 (0.0)	0 (0.0)	0 (0.0)	1 (0.4)	2 (0.7)	3 (1.1)	3 (0.5)	2 (0.3)	5 (0.8)	3 (0.5)	5 (0.8)	8 (1.2)	1 (0.4)	2 (0.9)	3 (1.3)
HPV45	0 (0.0)	0 (0.0)	0 (0.0)	0 (0.0)	2 (0.7)	2 (0.7)	2 (0.3)	1 (0.2)	3 (0.5)	8 (1.2)	2 (0.3)	10 (1.6)	0 (0.0)	1 (0.4)	1 (0.4)

#, number; HPV, human papillomavirus. Cases in which multiple HPV genotypes were detected were counted for each genotype, and so were counted more than once.

### 3.4 Proportion of HPV genotypes attributed to ICC according to histologic type and age

**Figure 2:**
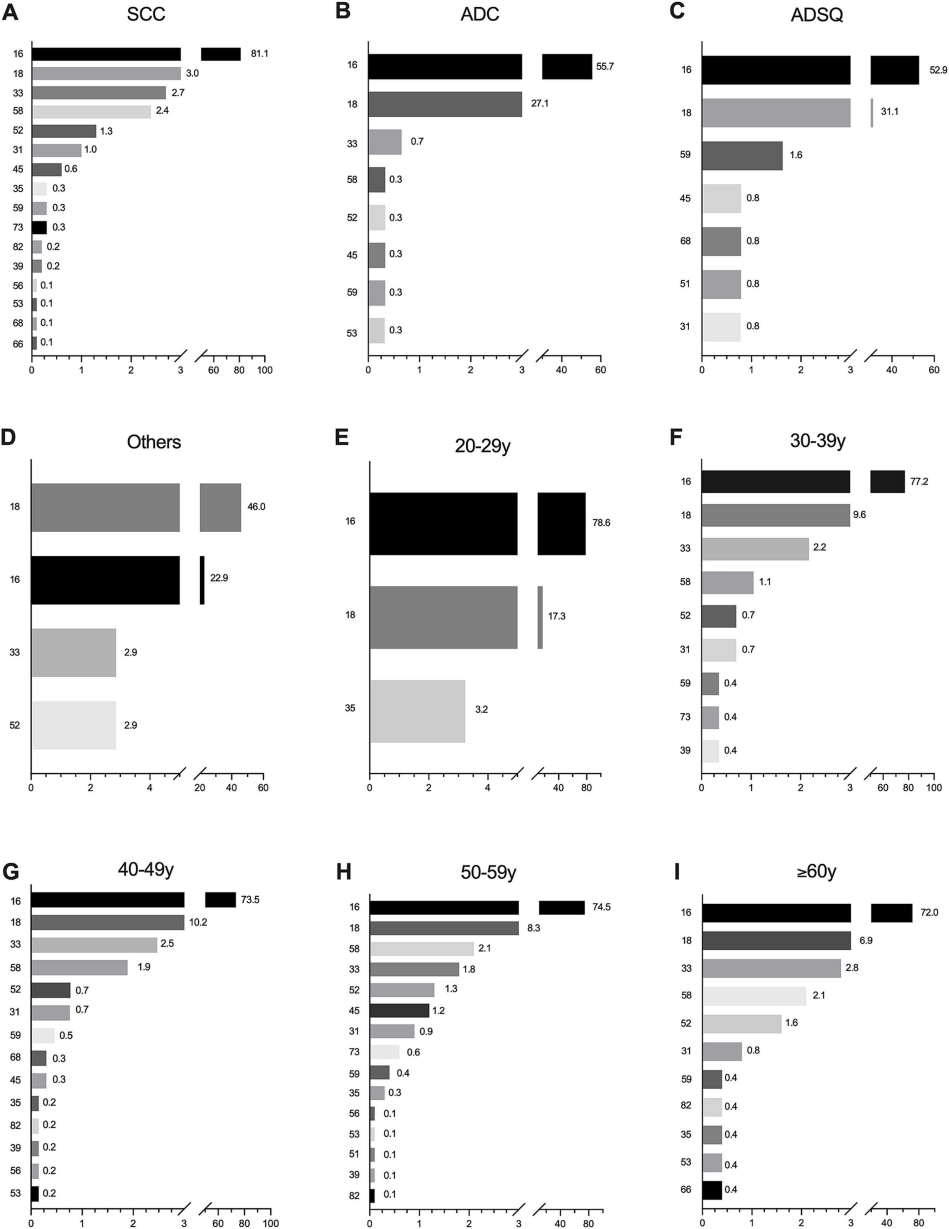
Attribution rates of common HPV genotypes by histological category and age group in invasive cervical cancer patients. **(A)** SCC; **(B)** ADC; **(C)** ADSQ; **(D)** Other rare histological categories; **(E)** 20–29 years; **(F)** 30–39 years; **(G)** 40–49 years; **(H)** 50–59 years; **(I)** ≥60 years. Only HPV genotypes with relative contributions greater than zero are shown. SCC, squamous cell carcinoma; ADC, adenocarcinoma; ADSQ, adenosquamous cell carcinoma; Others, other rare carcinomas.

In ICC, the most contributing HPV genotypes in SCC and ADC were HPV16, 18, 33, and 58, and the most contributing in ADSQ and other rare carcinomas were HPV16 and 18, followed by HPV52, 31, and 45 in SCC (Figure 2A); HPV52, 45, and 59 in ADC (Figure 2B); HPV59, 45, 51, 68, and 31 in ADSQ (Figure 2C); and HPV 33 and 52 in other rare carcinomas (Figure 2D). In the 20–29 age group, the most contributing were HPV16, 18, and 35 (Figure 2E); in the 30–49 and ≥60 age groups, HPV16, 18, 33, 58, 52, 31, and 59 were the seven most relatively contributing HPV genotypes (Figure 2F,G,I); and in the 50–59 age group, it HPV16, 18, 58, 33, 52, 45, and 31 were the most contributing HPV types (Figure 2H).

### 3.5 Vaccination coverage of bivalent and 9-valent HPV vaccines and optimized combinations of different HPV genotypes in ICC

**Figure 3:**
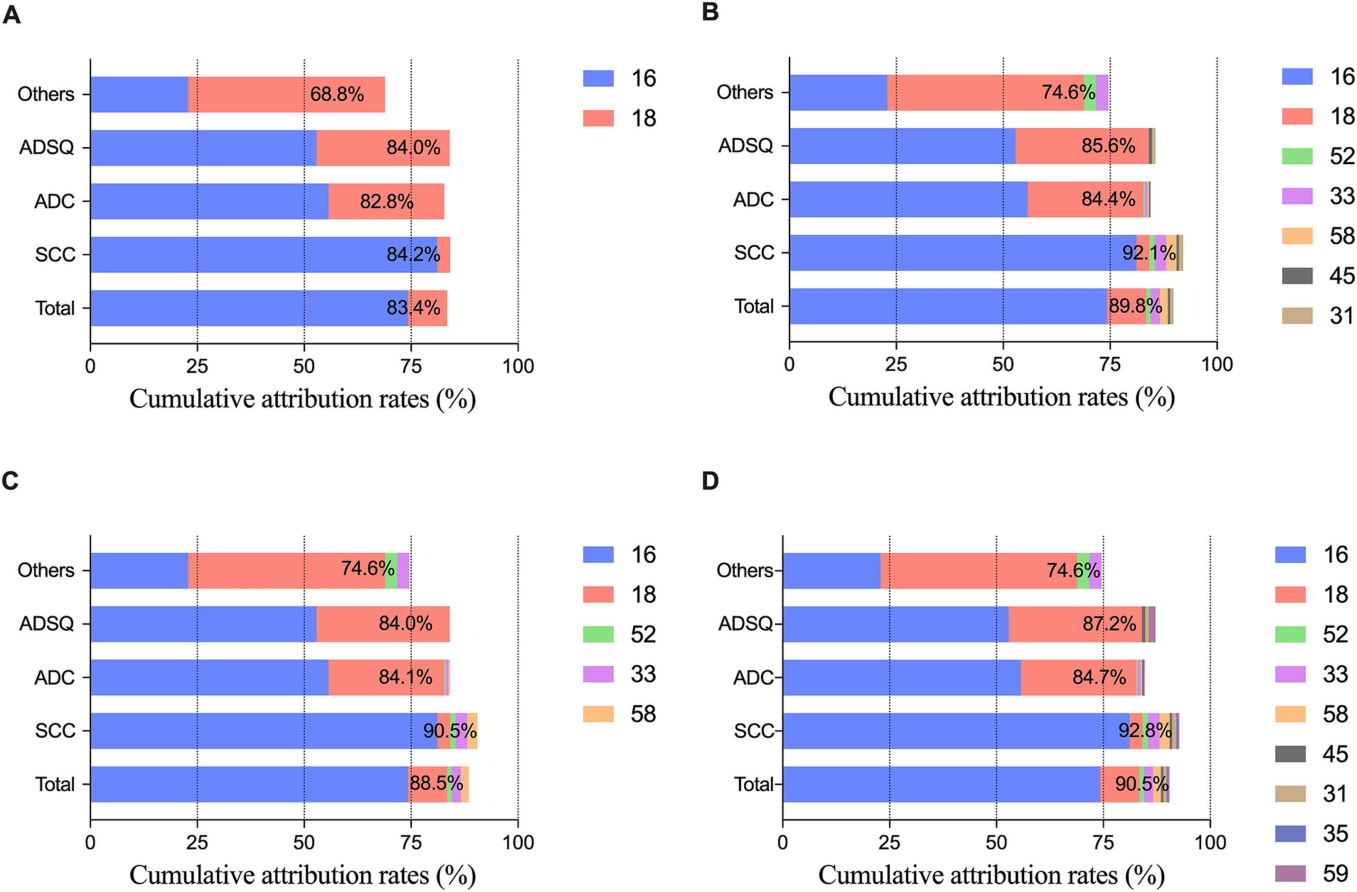
Cumulative attribution rates of different combinations of HPV genotypes according to histologic types. **(A)** Cumulative attribution rates of a bivalent vaccine composed of HPV16 and HPV18; **(B)** Cumulative attribution rates of HR-HPV in the 9-valent vaccine; **(C)** Cumulative attribution rates of optimization group A; **(D)** Cumulative attribution rates of optimization group B. SCC, squamous cell carcinoma; ADC, adenocarcinoma; ADSQ, adenosquamous cell carcinoma; Others, other rare carcinomas.

**Figure 4:**
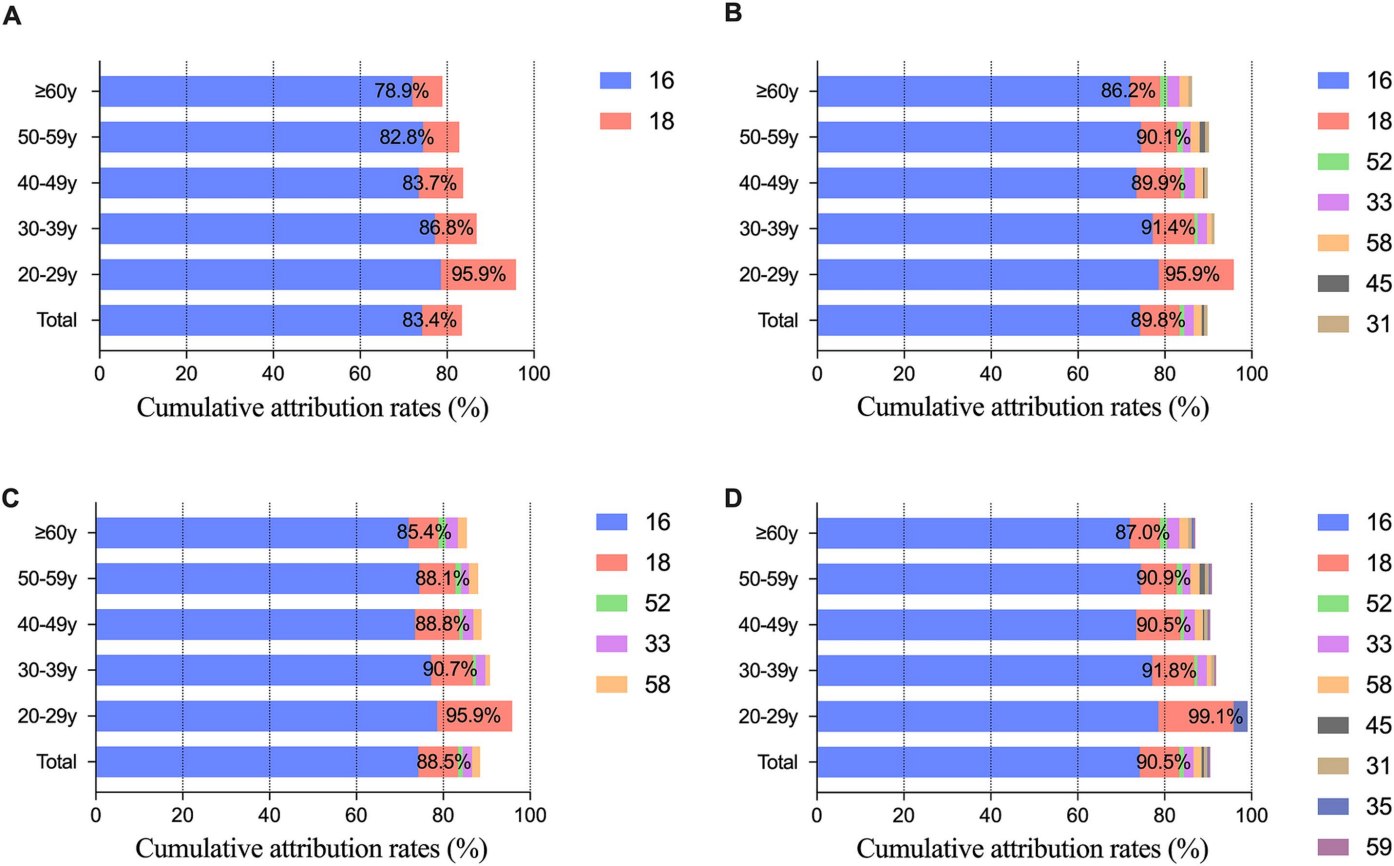
Cumulative attribution rates of different HPV genotypes combinations according to age group. **(A)** Cumulative attribution rates of the bivalent vaccine consisting of HPV16 and HPV18; **(B)** Cumulative attribution rates of HR-HPV in the 9-valent vaccine; **(C)** Cumulative attribution rates of optimization group A; **(D)** Cumulative attribution rates of optimization group B.

Through incidence and weighted attribution analysis, the quintet of HPV genotypes (HPV16, 18, 33, 52, and 58) with the most substantial contributions were categorized under optimization group A. Moreover, the top nine HPV genotypes (HPV16, 18, 31, 33, 35, 45, 52, 58, and 59) were assigned to optimization group B. In SCC, the cumulative attribution rates was 84.2% for the bivalent vaccine, 92.1% for the 9-valent vaccine, 90.5% for optimization group A, and 92.8% for optimization group B; in ADC, it was 82.8, 84.4, 84.1, and 84.7%; and in ADSQ and other rare cancers, it was 84.0, 85.6, 84.0, and 87.2% and 68.8, 74.6, 74.6, and 74.6%, respectively. The 9-valent vaccine increased the cumulative attribution rate in SCC by 7.9%, while the other three histological types increased the coverage by 1.6, 1.6, and 5.8%, respectively. The cumulative attribution rate of optimization group A increased by 1.3–6.3% compared with that of the bivalent vaccine. Optimization group B improved the coverage by 1.9 ~ 8.6% (Figure 3). The cumulative attribution rate for the four different HPV genotype combinations at different ages were as follows: 20–29 years, 95.9, 95.9, 95.9, and 99.1%; 30–39 years, 86.8, 91.4, 90.7, and 91.8%; 40–49 years, 83.7, 89.9, 88.8, and 90.5%; 50–59 years, 82.8, 90.1, 88.1, and 90.9%; and ≥ 60 years, 78.9, 86.2, 85.4, and 87.0% (Figure 4).

## 4 Discussion

Here, we provide reference data on the distribution of 23 HPV genotypes in paraffin-embedded tumor tissues of women with ICC in western China from 2018 to 2023. All samples in this study were collected after the introduction of the HPV vaccine in 2017 (12, 13), and HPV vaccination was still not subsidized by the government as of the completion of the study. Therefore, the data presented can be used as a baseline for monitoring when vaccination is widespread. These data confirm the prevalence of the five most common HPV genotypes (HPV16, 18, 33, 52, and 58) in ICC.

Our findings revealed that all detected single infection HPV genotypes were HR-HPV, constituting 80.2% of the overall infections; this result is consistent with the findings of Tang et al., who reported that the majority of women with squamous cervical lesions are infected with a single HPV genotype (14). In contrast, our results did not reveal any instances of single infections with LR-HPV, further confirming that HPV infections, especially HR-HPV infections, are the primary etiologic factor in cervical cancer (8). The HPV prevalence in our cohort is approximately 94.9%, which is comparatively high when contrasted with the rates among cervical cancer patients in other regions of China, ranging from 78.67 to 97.2% (11, 15–17). This discrepancy may be attributed to different detection methods’ varying capabilities in identifying specific HPV positivity, as Yin et al. (18) reported. Furthermore, the high HPV prevalence underscores the urgent need to intensify cervical cancer prevention initiatives in western China. HPV genotypes HPV16 and 18 were identified in 86.2% (1,645/1,908) of the patients, which is consistent with these being the most common oncogenic HPV genotypes worldwide (19), followed by HPV genotypes HPV58, 33, and 52. While, consistent with literature reports on geographical variations in HPV distribution, our study results differ from the predominant HPV genotype distributions reported in other regions of China (12, 15, 20–24). A potential reason for this discrepancy may be our inclusion criteria. The samples included in our analysis had HPV detection results from FFPE tumor tissues obtained after a definitive diagnosis of ICC, which contrasts with the majority of inclusion criteria that combine HPV diagnosis with cervical cytology and biopsy findings. Previous studies, including those by our colleagues Tang et al. (25), have established that HPV52 is the most prevalent infection genotype in the Chinese population (13, 26). Tang et al. (25) observed a decrease in HPV52 infection incidence with increasing lesion severity. Some investigators have suggested that fewer oncogenic HPV genotypes, such as HPV52, may persist longer at each stage of cervical carcinogenesis (27) and are slowly cleared during this process. In our analysis, HPV52 was detected in 2.2% of the patients, with 47.6% of these HPV52-positive patients coinfected with either HPV16 or HPV18. Consequently, we hypothesize that coinfection with the highly oncogenic HPV genotypes HPV16/18 may reduce the prevalence of HPV52 or even result in its elimination due to potential antagonistic interactions. HPV18, 16, and 45 have been reported to be the most common genotypes in adenocarcinomas worldwide (28). However, Chan et al. identified HPV18 as the second most prevalent genotype in adenocarcinoma patients, with HPV16 maintaining the first position (11), a finding consistent with our results. Additionally, we identified HPV33 as the next most common HPV genotype.

To explore trends in the prevalence of HPV infection over time, researchers categorized patients into five groups based on age. Wheeler et al. reported that women with ICC attributed to HPV16 or HPV18 infections were, on average, younger (27). This finding is corroborated by our results, which indicate that patients with ICC who tested positive for HPV16 or HPV18 had a mean age at diagnosis that was 1.69–2.53 years lower than that of patients who tested positive for other oncogenic HPV genotypes. Vinokurova et al. reported that HPV16, 18, and 45 more readily integrate into the human genome than other genotypes and that tumors may appear earlier during infection with these genotypes (29). In our study cohort encompassing individuals aged 20 to 29 years, 96.8% of patients had HPV16 or HPV18 infection, a finding that corroborates previous observations. However, this association was not observed for HPV45. Notably, the youngest individual in our cohort was a 21-year-old patient with a single HPV35 infection who reported engaging in first sexual intercourse at age 20 years and had no family history of cervical cancer. The literature indicates that HPV35 is genetically most closely related to HPV16 (30). This result raises the question of whether, akin to HPV16, HPV35 in young patients with a single infection might also more readily integrate into the human genome, potentially shortening the progression to invasive cancer. However, further data are necessary to substantiate this hypothesis due to the scarcity of epidemiological evidence. In contrast, the HPV35 infection rate in this study was 0.6%, which is consistent with the prevalence of HPV35 of approximately 2% in ICCs globally (30), which occurs only in SCCs and ADCs. Therefore, this genotype should also be considered in genotype-specific screening programs, providing additional surveillance opportunities for positive women.

In an inaugural large-scale survey of ICC patients in western China after the deployment of the HPV vaccine, we were surprised to discover that none of the patients had a history of HPV vaccination. This finding may correlate with the level of awareness regarding the vaccine and the initial age constraints imposed on its administration (31). China introduced the HPV vaccination in 2017; however, as of 2023, it remains excluded from the national immunization program (32). Moreover, the cost of the nine-valent vaccine is RMB 5800, approximately twice the amount charged in Western countries (26). This financial disparity can impose a heavier burden on some low-income populations (33, 34), a factor that aligns with the global trend where vaccination rates are positively correlated with higher income and education levels. Concurrently, our study revealed that the mean age at diagnosis of ICC was 49 years, which is lower than the global mean age at diagnosis for cervical cancer, which is 53 years (1). Consequently, it is imperative for government intervention programs to prioritize the reduction of the HPV-related cervical cancer burden (35).

The suitability of HPV vaccination for populations, once predominantly determined by age and the presence of sexual activity, is seeing diminishing restrictions based on sexual history as the age range for vaccination expands (5, 31, 33, 36), and all patients in our cohort had a history of sexual behavior. We attributed multiple infections to a single HPV genotype using weighted attribution ratios to determine the contribution of each genotype across different age groups and histologic types. Our analysis indicated that the overall potential vaccination coverage of the bivalent vaccine was 83.4%. In contrast, the coverage of genotypes A and B in our optimized group was very close to that of the nine-valent vaccine, at 88.5, 90.5 and 89.8%, respectively. Among the various histological types, attribution to other rare carcinomas was exclusively associated with HPV genotypes HPV16, 18, 33, and 52. This association resulted in a consistent coverage of 74.6% for both the nine-valent vaccine and our optimized groups A and B, which was approximately 5.8% greater than that of the bivalent vaccine. In the case of ADSQ, the highest coverage was observed in optimized group B, which surpassed the consistent coverage of the bivalent vaccine and optimized group A by approximately 3.2% and that of the nine-valent vaccine by approximately 1.6%, respectively. The coverage of the bivalent vaccine to ADC was 82.8%, which was 1.3 to 1.9% lower than that of the other three HPV vaccine combinations. For SCC, the most prevalent histological type, the coverage in optimization group A was 90.5%, indicating a 6.3% improvement over the bivalent vaccine and a 1.6–2.3% decrease compared to the nine-valent vaccine and optimization group B. The efficacies of both the bivalent and nine-valent vaccines observed within our study were greater than those reported by Chan et al. (11) in Hong Kong, China, for cervical squamous carcinoma (59.5 and 76.6%, respectively) and adenocarcinoma of the cervix (78.6 and 80.6%, respectively). The same trend was observed in Shandong Province, China, for cervical squamous cell carcinoma (49.1 and 73.1%, respectively) and adenocarcinoma of the cervix (65.1 and 69.3%, respectively) (17). The reason may be that we used tumor tissue for HPV testing after ICC diagnosis.

Both the bivalent and 9-valent vaccines, as well as optimized group A, exhibited a potential vaccination coverage of 95.9% in the 20–29 years age group. This result is attributable to the exclusive association of this demographic with HPV genotypes HPV16, 18, and 35. Therefore, the inclusion of HPV35 in optimized group B led to a coverage of 99.1%. In the 30- to 59-year-old age group, the 9-valent vaccine and optimized group B had similar coverage, each providing approximately 90%, while optimized group A provided more than 88%. In contrast, the coverage of the bivalent vaccine was slightly lower, at just above 83%. Among patients aged 60 years and older, the coverage of optimized group A was 85.4%, which was 6.5% greater than that of the bivalent vaccine and 0.8–1.6% less than that of the other two HPV combination vaccines. Regardless of the HPV vaccine combination, however, vaccine protection gradually declined with age.

HPV vaccines currently available in China include two domestic bivalent HPV vaccines (Cecolin, Xiamen Innovax Biotech Co., Ltd.; Walrinvax, Yuxi Walvax Biotech Co., Ltd.) and several foreign HPV vaccines (Cervarix, Gardasil^®^, and Gardasil-9^®^) that have not been formally included in the national vaccination. The literature indicates that the protective efficacy of the HPV vaccine is generally sustained for 6–11 years postvaccination (37, 38). Although the age range for vaccination has been extended to 45 years (39), administering the vaccine prior to the initiation of sexual activity is considered to maximize cost-effectiveness (40). Vaccination also enhances herd immunity and provides cross-protection against various HPV genotypes (36). Our findings show that the bivalent vaccine has a potential vaccination coverage of 83.4%, which exceeds the reported 79% in Sweden (41). This, in conjunction with the perspectives of national researchers (26, 34), suggests that prioritizing the inclusion of the bivalent vaccine in the national free immunization program is the most economically efficient strategy. In western China, if an optimized vaccine strategy, such as optimized group A, is considered, the vaccine coverage in this region could approach that of the 9-valent vaccine, thereby reducing the economic burden on the vaccinated population. The 9-valent vaccine and optimized group B could be options for those who are financially capable, which is the most beneficial choice for the public. Our study is not only fairly representative of the HPV infection patterns in Western China and could add to the already existing body of evidence regarding other Chinese regions but also can aid policymakers in maximizing cost-effectiveness. This information enables the public to make informed choices regarding various HPV vaccine combinations when their financial means allow, thereby contributing to the objective of enhancing women’s health and mitigating the social and economic impact of cervical cancer.

Our study has several limitations. First, although our study population was derived from the western provinces of China, the demographic distribution of our data may have been influenced by the COVID-19 outbreak and the policies implemented to manage the epidemic during data sourcing. Thus, as in all retrospective studies from a single institution, selection bias was present. Second, the small sample size of patients with other rare carcinomas and patients in the younger age group could have resulted in a bias toward attributing the results to a limited number of HPV genotypes. Third, since none of the enrolled ICC patients were vaccinated, our results are speculative and will require further evaluation of the vaccinated population in the future. However, our study also possesses significant strengths. First, we analyzed HPV detection in FFPE tumor tissues from patients who were diagnosed with ICC following primary radical surgery. This represents one of the largest studies conducted in this manner to date in western China and one of the most comprehensive in-depth analyses of age-related factors. Additionally, we employed a sensitive polymerase chain reaction (PCR) reverse dot hybridization technique complemented by the use of internal control (IC) sites to enforce stringent internal quality control measures. This study further strengthens the rationale for utilizing existing vaccines for the prevention of cervical cancer and provides insights to inform future optimization of vaccination strategies.

## 5 Conclusion

To our knowledge, this study represents the most comprehensive analysis of HPV infection results derived from paraffin-embedded tumor tissue samples for histologic type and age stratification following the diagnosis of ICC in western China. Our findings indicate that the incorporation of a bivalent vaccine into the national immunization program at the governmental level is the most cost-effective strategy. Furthermore, our study suggested that optimization group A closely approximates the coverage of the 9-valent vaccine while concurrently reducing the financial burden on the vaccinated population. Additional combinations of HPV genotypes are accessible to those with the financial means to opt for them. As awareness of the HPV vaccine proliferates, we anticipate that these findings will serve as a guide for future cervical cancer prevention initiatives in western China.

## Data Availability

The original contributions presented in the study are included in the article/Supplementary material, further inquiries can be directed to the corresponding authors.
