# Supplementary material for: HPV genotypes in invasive cervical cancer: prevalence, risk attribution, and optimized vaccine strategies in western China
Source: Front Public Health. 2024 Dec 18;12:1455931. doi: 10.3389/fpubh.2024.1455931 (PMC11688338; doi:10.3389/fpubh.2024.1455931)

| **Supplementary** **Table S1**  Distribution of HPV genotypes in different histologic types invasive cervical cancer. | | | | | | | | | | |
| --- | --- | --- | --- | --- | --- | --- | --- | --- | --- | --- |
| HPV type | SCC(n=1436) | | ADC(n=308) | | ADSQ(n=129) | | Others(n=35) | | total(n=1908) | |
|  | single | ≥2 types | single | ≥2 types | single | ≥2 types | single | ≥2 types | single | ≥2 types |
| 16 | 1008 | 185 | 148 | 36 | 54 | 25 | 6 | 7 | 1216 | 253 |
| 18 | 41 | 70 | 72 | 36 | 35 | 14 | 13 | 5 | 161 | 125 |
| 31 | 14 | 10 | 0 | 0 | 1 | 1 | 0 | 0 | 15 | 11 |
| 33 | 38 | 35 | 2 | 1 | 0 | 0 | 1 | 0 | 41 | 36 |
| 35 | 5 | 4 | 0 | 2 | 0 | 0 | 0 | 0 | 5 | 6 |
| 39 | 3 | 2 | 0 | 0 | 0 | 0 | 0 | 0 | 3 | 2 |
| 45 | 8 | 3 | 1 | 1 | 1 | 2 | 0 | 0 | 10 | 6 |
| 51 | 0 | 14 | 0 | 1 | 1 | 2 | 0 | 0 | 1 | 17 |
| 52 | 18 | 18 | 1 | 1 | 0 | 3 | 1 | 0 | 20 | 22 |
| 53 | 2 | 5 | 1 | 0 | 0 | 1 | 0 | 0 | 3 | 6 |
| 56 | 2 | 7 | 0 | 3 | 0 | 1 | 0 | 0 | 2 | 11 |
| 58 | 33 | 38 | 1 | 3 | 0 | 3 | 0 | 1 | 34 | 45 |
| 59 | 5 | 4 | 1 | 1 | 2 | 5 | 0 | 1 | 8 | 11 |
| 66 | 1 | 1 | 0 | 0 | 0 | 0 | 0 | 0 | 1 | 1 |
| 68 | 1 | 4 | 0 | 1 | 1 | 2 | 0 | 0 | 2 | 7 |
| 73 | 5 | 2 | 0 | 0 | 0 | 0 | 0 | 0 | 5 | 2 |
| 82 | 3 | 3 | 0 | 0 | 0 | 0 | 0 | 0 | 3 | 3 |
| 6 | 0 | 4 | 0 | 0 | 0 | 0 | 0 | 0 | 0 | 4 |
| 11 | 0 | 6 | 0 | 0 | 0 | 0 | 0 | 0 | 0 | 6 |
| 42 | 0 | 2 | 0 | 1 | 0 | 0 | 0 | 0 | 0 | 3 |
| 43 | 0 | 4 | 0 | 2 | 0 | 0 | 0 | 0 | 0 | 6 |
| 81 | 0 | 4 | 0 | 0 | 0 | 0 | 0 | 0 | 0 | 4 |
| 83 | 0 | 1 | 0 | 0 | 0 | 0 | 0 | 0 | 0 | 1 |

HPV, human papillomavirus; SCC, squamous cell carcinoma; ADC, adenocarcinoma; ADSQ, adenosquamous cell carcinoma; Others, other rare carcinomas.

| **Supplementary Table S2**  HPV distribution in cases of invasive cervical cancer by age. | | | | | |
| --- | --- | --- | --- | --- | --- |
|  | cases# | HPV-negative#(%) | HPV- positive#(%) | Single HPV#(%) | Multiple HPV #(%) |
| 20-29y | 31 | 0(0.0) | 31(100) | 28(90.3) | 3(9.7)) |
| 30-39y | 288 | 13(4.5) | 275(95.5) | 228(79.2) | 47(16.3) |
| 40-49y | 660 | 34(5.2) | 626(94.8) | 534(80.9) | 92(13.9) |
| 50-59y | 676 | 32(4.7) | 644(95,3) | 549(81.2) | 95(14.1) |
| ≥60y | 253 | 19(7.5) | 234(92.5) | 191(75.5) | 43(17.0) |
| Total | 1908 | 98(5.1) | 1810(94.9) | 1530(80.2) | 280(14.7) |

#, number;HPV, human papillomavirus;

**Supplementary Figure 1. Geographic distribution of sample sources for the 1908 cases in this study.**


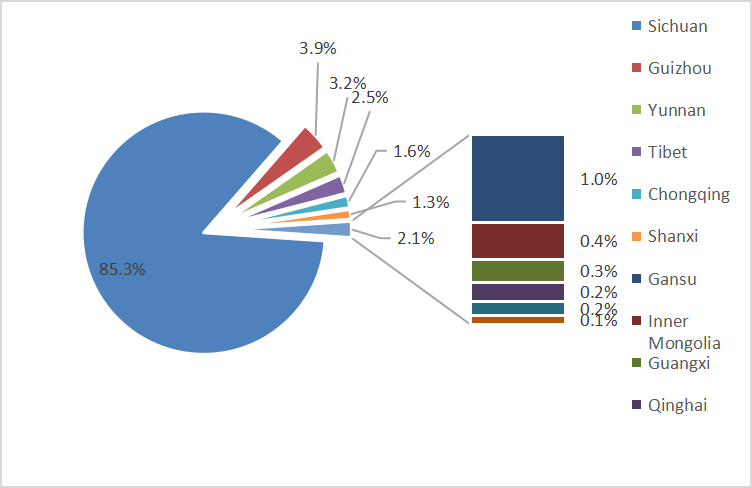

Supplement: Supplementary file 1 [file Data_Sheet_1.doc]
